# Theoretical Perspectives on Hookah Smoking Behavior: A Scoping Review of Studies Among Young Adults in Low- and Middle-Income Countries

**DOI:** 10.1007/s10900-025-01449-7

**Published:** 2025-03-06

**Authors:** Christina Asiedua, Elakeche Abah, Matthew Asare

**Affiliations:** Department of Public Health, Robbins College of Health and Human Sciences Baylor University, One Bear Place 97343, Waco, TX 76798 USA

**Keywords:** Hookah, Shisha, Waterpipe, Low-to-middle income countries, Theory, Young adults

## Abstract

Hookah smoking among young adults is a growing public health concern, especially in low- and middle-income countries (LMICs). Despite its prevalence, comprehensive reviews evaluating theoretical frameworks used to study this behavior are lacking. This scoping review synthesized studies examining hookah smoking behaviors among young adults in LMICs. A systematic search was conducted in PubMed, Embase, Google Scholar, and CINAHL using keywords such as “Hookah OR Waterpipe OR Shisha,” “Adverse effects OR Harmful Effects,” “Young adults OR Adolescents,” “Theory,” and “Low-middle-income countries.” Articles meeting predefined inclusion and exclusion criteria were screened using Covidence, and data on prevalence, predictors, and theoretical frameworks were extracted. The review included 28 studies involving 13,150 young adults with an average age of 19.2 years. Hookah use prevalence ranged from 2.6 to 89.4%, with a pooled prevalence of 26.4%. Frequently applied theoretical frameworks included the Theory of Planned Behavior (TPB) and Social Cognitive Theory (SCT), along with the Protection Motivation Theory and PRECEDE-PROCEED. Facilitators of hookah use included subjective norms, favorable attitudes, perceived low risk, environmental influences, and limited awareness of health risks. Protective factors included self-efficacy, perceived risks, awareness of harmful effects, and anti-smoking attitudes. Interventions based on TPB and SCT showed positive outcomes, though some failed, highlighting the need for culturally sensitive approaches. The findings emphasize the importance of education and policy measures targeting both individual and environmental factors to reduce hookah smoking prevalence and its associated risks in LMICs.

## Introduction

Tobacco smoking, though preventable, remains the leading cause of premature death worldwide, accounting for over 8 million deaths annually [1]. This burden disproportionately affects low- and middle-income countries (LMICs) [1]. Tobacco is consumed in various forms, including cigarettes, snuff, chewing tobacco, and hookah. Historically, hookah—also known as waterpipe, shisha smoking—has been used for centuries in regions such as the Eastern Mediterranean, the Middle East, and parts of Asia [2].

In recent years, hookah smoking has become a global social trend, heavily marketed as a safer, non-addictive alternative to cigarettes [3, 4]. The growing prevalence of hookah use, and its associated health risks make it a critical issue for the public health community. Hookah smoking is associated with numerous adverse health outcomes, including oral cancer, other cancers, cardiovascular diseases, asthma, chronic obstructive pulmonary disease, and poor oral health [5, 6]. Like other tobacco products, hookah smoking contributes significantly to disease, disability, and premature death worldwide [7]. Recent studies highlight that hookah smoking can lead to nicotine addiction comparable to or even greater than cigarette smoking [8].

Hookah smoking, while common across various age groups, has gained significant popularity among young adults, reaching near-epidemic levels [9]. This trend is particularly pronounced among adolescents and college students, driven by the widespread availability of flavored hookah tobacco and its appealing taste and aroma [10, 11]. Young adults from diverse ethnic backgrounds are especially drawn to hookah due to its perception as a safer and more socially acceptable alternative to cigarettes [12, 13]. In Palestine, for instance, hookah smoking rates are as high as 35–56% among university students [14].

Several factors influence hookah smoking among youth. A prevalent misconception is that it is less harmful than cigarettes. Young people often turn to hookah for fun, stress relief, and as a means of expressing masculinity or maturity [7, 15]. Additional motivations include curiosity, boredom, pleasure-seeking, and a lack of awareness about health risks. Sweet, flavored tobacco, the belief that hookah is non-addictive, and its perception as a temporary and socially accepted habit also contribute to its growing popularity [2, 16, 17]. Addressing the misconceptions and targeting the factors that drive hookah use are essential steps toward reducing its impact on global health.

The World Health Organization (WHO) highlights that research on hookah’s health impacts remains underexplored and calls for further investigation to better understand its cancer risks and develop preventive measures [5]. Although some theoretical frameworks have been used to examine the health effects of hookah smoking, there is a lack of comprehensive reviews evaluating these studies. Conducting a comprehensive review of theory-based studies to understand the health implications of hookah use is important because it provides a deeper insight into how different behavioral, social, and psychological factors influence hookah smoking. This approach helps to integrate existing knowledge, identify gaps in the research, and guide the development of more effective, evidence-based interventions and public health policies aimed at reducing the associated health risks. This scoping review aimed to provide a comprehensive summary of theory-based studies examining hookah smoking behaviors in low- and middle-income countries over the past decade.

## Methods

### Information Sources and Search Strategy

A systematic search was conducted between August 2022 and July 2024 across several databases, including PubMed, Embase, Google Scholar, and CINAHL to identify relevant studies. The focus was on studies examining the burden, factors, and predictors of hookah (waterpipe) smoking among adolescents and young adults aged 10–30 years. The search used a combination of keywords: *“Hookah OR Waterpipe OR Shisha”* AND *“Cancer OR Cancer Risks OR Risks of Cancer”* AND *“Young Adults OR Youth OR Adolescents”* AND *“Theoretical Framework OR Constructs OR Theory OR Model”* AND *“Low-Income Countries OR Low-to-Middle-Income Countries.”* Articles published in English and peer-reviewed journals between 2014 and 2024 that met these criteria were included. Covidence software was used to streamline the process by screening articles, removing duplicates, and extracting data efficiently.

### Eligibility Criteria and Study Selection

**Fig. 1:**
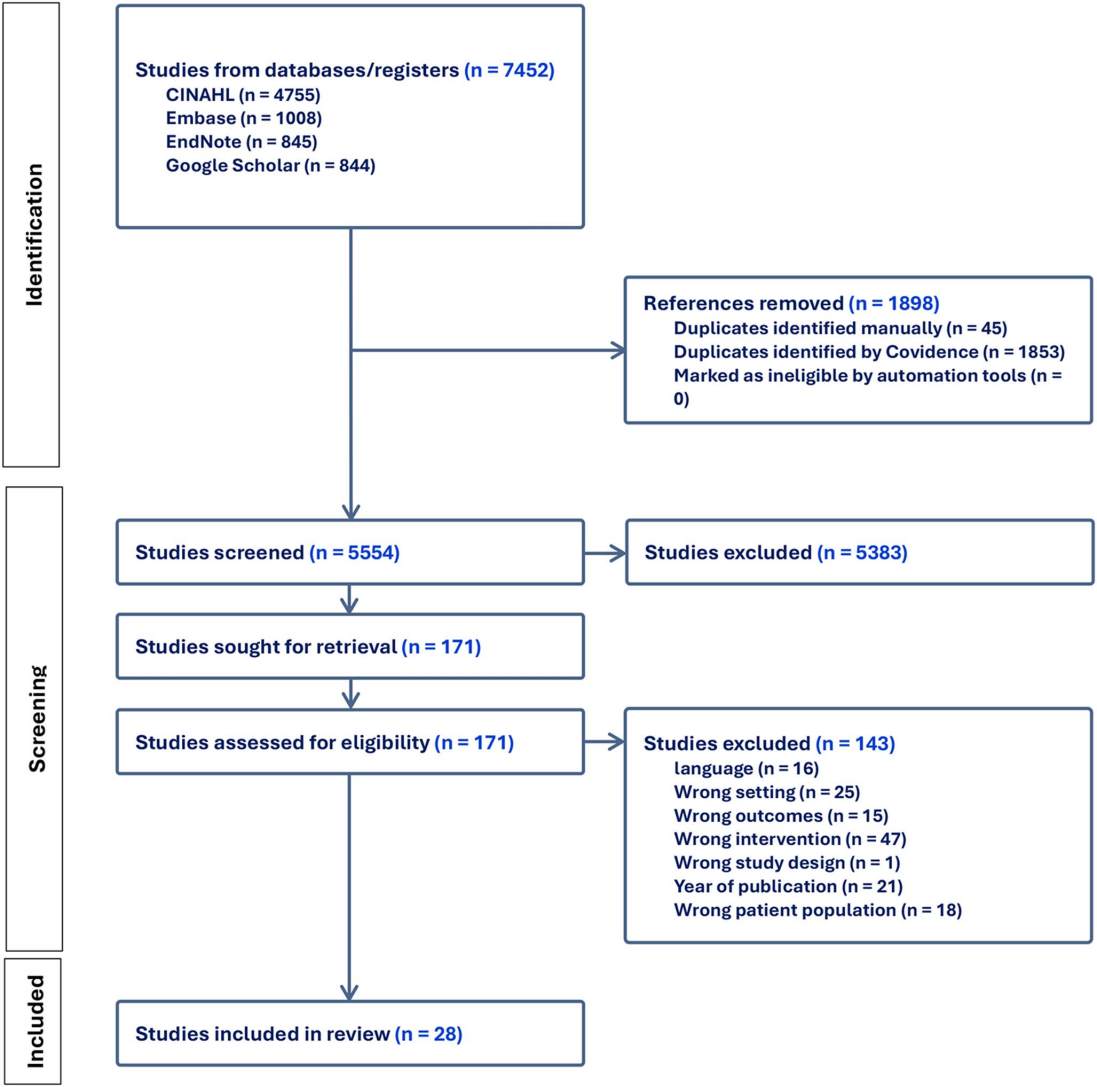
PRISMA diagram of the summary of articles screened and extracted

This scoping review utilized specific criteria to identify relevant articles. Study designs such as randomized control trials, quasi-experimental designs, interventional studies, cross-sectional, and qualitative (i.e., interviews and focus group discussions) studies were included, while review articles including meta-analyses, scoping and systematic reviews were excluded. Articles were included in the review if they met the following criteria: (a) focused on hookah, waterpipe, or shisha smoking; (b) involved participants aged 10 to 33 years; (c) included high or middle school students, college students, or community-based youth and young adults; (d) incorporated a theoretical framework, model, or one or more constructs of a theory; (e) were conducted in low-to-middle-income countries; (f) were published in English; and (g) were electronically accessible. In addition, articles focusing solely on substance use and cigarette smoking were excluded, and articles that studied only the prevalence of hookah smoking were also excluded. Articles that did not utilize theoretical frameworks, models, or any constructs in the study were also excluded. Articles that included participants over 30 years old and conducted in high income countries were excluded. Only peer-reviewed articles were considered; the thesis, discussion papers, and editorials were excluded. Two independent reviewers (C.A. and J. L.) reviewed the articles. These reviewers independently screened the title, abstract, and full text and occasionally met to discuss and resolve any conflicts in the screening to ensure that the inclusion criteria were consistently followed. The senior researcher on the team (M.A.) supervised all the screening and ensured that all inclusion criteria were met. The initial search identified 7,452 articles. After removing duplicates using Covidence, the number of unique articles was reduced to 5,554. Titles and abstracts were screened to identify articles potentially meeting the inclusion criteria, narrowing the selection to 171 for full-text review. Figure 1 shows a PRISMA diagram summarizing the selection process.

### Data Extraction

Data extraction was performed systematically using Covidence to organize the review process. Relevant information, including the year of publication, country, setting, sample size, study design, theoretical framework or constructs, and primary findings, was independently extracted by both reviewers. Any discrepancies were resolved through discussion or consultation with a third (senior researcher) reviewer. The extracted data were compiled into summary tables for analysis, focusing on the study objectives, theoretical frameworks, and findings on hookah smoking behavior in low- and middle-income countries.

## Results

### Study Characteristics

After reviewing the full texts, 28 articles met the inclusion criteria and were thus included in the review. Table 1 provides a summary of all included articles. These 28 studies collectively involved 13,150 teenagers and young adults, with an average age of 19.2 years. Among the participants, 6,425 (48.8%) were male, 5,802 (44.1%) were female, and 1,220 (9.3%) did not have a reported gender. The studies were conducted across six countries, 22 of which were conducted in Iran [5, 7, 18, 22, 23–32, 35, 36, 38–42, 43], two in Saudi Arabia [20, 37], and one study was conducted in each of the following countries; Indonesia [21], Pakistan [34], Ethiopia [19], and Uganda [33]. Sample sizes varied widely, ranging from 24 participants [31] to 3,347 participants [19]. Many of the studies (*n* = 18) were conducted in educational settings [7, 18–43, 23–30, 35, 36, 20, 40–33]; three were conducted in health centers [5, 43, 41], and the remaining seven were carried out in urban areas, such as community coffee shops and drinking bars [31, 32, 32–40, 42, 37].

**Table 1:** The summary of articles (*n* = 28) included in the review

Rahimi & Javadi, 2018 [18]	Purpose	Using Prototype Willingness Model to Predict Waterpipe Smoking among High School Adolescents.
	Country	Iran
	Study Design	Cross-sectional study
	Setting	High schools in Birjand
	Time Period	One year
	Population Description	High school students from grade 10 to grade 12
	Sample Size	432, Male = 239 Female = 193
	Outcome	Intention of hookah use
	Theory/Model/ Constructs	Prototype Willingness Model
	Primary Findings	i. Subjective norms and attitudes showed a positive correlation towards hookah smoking. ii. Belief that hookah smoking has lower risks than cigarettes increases their willingness to engage in the behavior. iii. Behavioral intention and behavioral willingness were significant predictors of hookah smoking among middle school adolescents. iv. Prevalent of Hookah smoking was 17.4%
Hirpa et al., 2021 [19]	Title	An Emerging Problem of Shisha Smoking among High School Students in Ethiopia.
	Country	Ethiopia
	Study Design	Cross-sectional study
	Setting	High Schools in Oromia, Amhara, Tigray, Addis Ababa
	Time Period	Not specified
	Population Description	Secondary school students, ranging in age from 13 to 22 years old, who were in grades 9 and 10.
	Sample Size	3347, Male = 1515 Female = 1804
	Outcomes	Hookah use behavior and perceptions about smoking
	Theory/Model/ Constructs	Subjective norms, environment, knowledge, beliefs
	Primary Findings	i. Students were aware of hookah smoking and its potential health effects. ii. Subjective norms (family or friends who smoke) affect students' attitudes toward it. iii. Environment, (i.e., proximity of hookah establishments to school campuses) influenced their inclination towards smoking hookah. iv. Prevalence of hookah use 2.6% more male vs. female
Almogbel et al., 2021 [20]	Title	Predictors of waterpipe smoking among university students in the Qassim region, Saudi Arabia.
	Country	Saudi Arabia
	Study Design	Cross-sectional study
	Setting	Higher education institutions in the Qassim region.
	Time Period	Five months
	Population Description	College students between the ages of 18 and 26 years.
	Sample Size	928, Male = 820 Female = 105
	Outcome	Hookah use behavior
	Theory/Model/ Constructs	Used constructs: Knowledge, attitude, subjective norms
	Primary Findings	i. Perception that hookah is a safer and a non-addictive alternative leads to smoking a hookah. ii. Subjective norms (family or friends who smoke) affect students' attitudes toward it. iii. Prevalent of hookah smoking 19.94% (more males vs female
Fauzi & Areesantichai, 2022 [21]	Title	Determinants of waterpipe smoking among high school students in Jakarta, Indonesia.
	Country	Jakarta, Indonesia
	Study Design	Cross-sectional study
	Setting	Public high Schools in Jakarta
	Time Period	One month
	Population Description	High school students between the ages of 15 and 19.
	Sample Size	1,318, Male = 828 Female = 490
	Outcomes	Hookah use behavior
	Theory/Model/ Constructs	Constructs: Subjective norms, environment
	Primary Findings	i. Friends or family members' hookah smoking is associated with individual hookah smoking behaviors. ii. Environment influences the attitudes of participants towards this activity. iii. Prevalent of hookah smoking is 5.4% (more males vs females)
Joveini et al., 2020 [22]	Title	The effects of an education program on hookah smoking cessation in university students: an application of the Health Action Process Approach (HAPA).
	Country	Iran
	Study Design	Quasi-intervention study The intervention comprised two parts: the motivational and the volitional sessions
	Setting	Islamic Azad University of Sabzevar.
	Time Period	Two years eight months
	Population Description	Undergraduate male students between the age of 19–25 years.
	Sample Size	137 (Male)
	Outcome	Intention to quit smoking and actual quitting of smoking
	Theory/Model/ Constructs	Health Action Process Approach (HAPA)
	Primary Findings	i. Perception that hookah smoking is safer and without health consequences increases its usage. ii. The sweet flavors and social introductions to the activity by friends also contributed to its appeal. iii. Decrease in the participants' intentions to reduce or quit hookah smoking. iv. Quit: Intervention group 44.1% vs. Control Group 9.4% v. Prevalent of hookah smoking was ~ 64%
Khani Jeihooni & Afzali Harsini, 2020 [23]	Title	The Effect of an Educational Intervention Based on PRECEDE Model on Oral Cancer Prevention Behaviors in Hookah Users.
	Country	Iran
	Study Design	Intervention (Intervention group 180 and control group 180)
	Setting	University campuses at Fasa
	Time Period	Not specified
	Population Description	College students between the ages of 18 and 33 years.
	Sample Size	360, Male = 147 Female = 213
	Outcome	Oral cancer prevention
	Theory/Model/ Constructs	PRECEDE-PROCEED
	Primary Findings	i. Positive attitude towards smoking because hookah is perceived as non-addictive and enjoyable. ii. The decision to quit was significantly influenced by the views of family and friends. iii. A positive attitude toward smoking diminished the inclination to quit hookah use. iv. Prevalent of hookah smoking was 32.3%
Sadeghi et al., 2019 [24]	Title	Predictive factors for preventing hookah smoking and health promotion among young people based on the protection motivation theory.
	Country	Iran
	Study Design	Cross-sectional study
	Setting	Two health centers in Sirjan, Iran
	Time Period	Not specified
	Population Description	Adolescents between the ages of 12 and 24 years.
	Sample Size	280, Male = 26 Female = 154
	Outcome	Factors Preventing hookah smoking
	Theory/Model/ Constructs	Protection motivation theory
	Primary Findings	i. Link between perceived susceptibility, severity, fear, and the prevention of hookah smoking. ii. Students who possessed sufficient awareness of the harmful health consequences of hookah smoking and harbored fears of life-threatening outcomes were less likely to partake in the activity. iii. Prevalent of hookah smoking was 35%
Rajabalipour, Khoshab, et al., 2019 [25]	Title	Using Social Cognitive Theory to investigate the risk factors of waterpipe smoking among Southeastern Iranian Adolescents.
	Country	Iran
	Study Design	Quasi-experimental design
	Setting	High Schools in Kerman, Sistan, and Baluchistan provinces.
	Time Period	Four months
	Population Description	High school students in grade tenth, eleven, and twelve grades with ages between 14 to 18 years.
	Sample Size	1196, Male = 623 Female = 595
	Outcome	Knowledge, self -efficacy to prevent hookah smoking.
	Theory/Model/ Constructs	Socio-Cognitive Theory i. Knowledge ii. Perceived self-efficacy iii. Outcome expectations iv. Environment v. Subjective norms, environment, knowledge
	Primary Findings	i. Adequate awareness about hookah smoking and its harmful effects. However, students scored low on self-efficacy skills, increasing their tendency to engage in the behavior. ii. Environmental factors of the students increased their hookah smoking behavior as there were fewer recreational facilities, sports facilities, and easy access to hookah. iii. The prevalence of hookah smoking was ~ 43.8% in the intervention group.
Momeni et al., 2019 [26]	Title	Frequency of waterpipe smoking and its effective factors among students of state universities in Kerman.
	Country	Iran
	Study Design	Cross-sectional study
	Setting	University and medical colleges in southeastern Iran.
	Time Period	Not specified
	Population Description	College students between the ages 10–21
	Sample Size	675, Male = 268 Female = 407
	Outcome	Hookah smoking behavior
	Theory/Model/ Constructs	Subjective norms, environment, knowledge
	Primary Findings	i. The most important reason for the waterpipe was entertainment, followed by curiosity. ii. Swayed by the attitudes of peers and family. iii. Prevalent of hookah smoking is 30.2% (more males vs females)
Karimy et al., 2015 [27]	Title	An Extended Theory of Planned Behavior (TPB) Used to Predict Smoking Behavior Among a Sample of Iranian Medical Students.
	Country	Iran
	Study Design	Cross-sectional study
	Setting	University campus
	Time Period	Not specified
	Population Description	University medical students between the ages of 18 and 25 years.
	Sample Size	170, Male = 114 Female 56
	Outcome	Intention to smoke hookah
	Theory/Model/ Constructs	Theory of planned behavior
	Primary Findings	i. Normative beliefs (friends and family who partake in smoking hookah) affect their disposition to engage in smoking hookah. ii. Students who had never smoked demonstrated the ability to resist peer pressure and refused to smoke. iii. Prevalent of hookah smoking is 83.4% (more males vs females)
Bashirian et al., 2019 [28]	Title	Water Pipe Smoking Reduction in the Male Adolescent Students: An Educational Intervention Using Multi-Theory Model.
	Country	Iran
	Study Design	Randomized Controlled Trial (Intervention Group vs. Control Group) The educational intervention using discussions, video and flyers.
	Setting	High schools in Hamadam City in Western Iran.
	Time Period	Three months
	Population Description	High school male students from grades 10–11 between the ages of 15 to 17.
	Sample Size	94 (Male)
	Outcome	Number of hookah use
	Theory/Model/ Constructs	Multi Theory Model
	Primary Findings	i. The participatory dialogue decreased hookah smoking behavior in the experimental group vs. control group. ii. Behavioral confidence influenced motivation and self-efficacy, diminishing the willingness to initiate the behavior, refuse offers from friends, and cease the behavior if already involved. iii. Intervention successfully changed the experimental group perceptions of hookah smoking into motivation and goal-setting strategies to maintain their reluctance to partake in the behavior iv. Reductions in the frequency of hookah use from 14.9–4.3% in the intervention group vs. From 8.5% 12.8% v. Prevalent of hookah smoking was 89.4% in the intervention group.
Bashirian et al., 2021 [29]	Title	The Effect of a Web-based Educational Program on Prevention of Hookah Smoking among Adolescent Girls: Application of Theory of Planned Behavior.
	Country	Iran
	Study Design	Randomized control trial Web-based educational intervention, followed by WhatsApp messages
	Setting	High school in Kermanshah city.
	Time Period	Not specified
	Population Description	Female high school between 14 and 18 years for both intervention and control groups.
	Sample Size	110 (Female)
	Outcomes	Intention and self-efficacy to resist smoking
	Theory/Model/ Constructs	Health Belief Model i. Attitude ii. Subjective norms iii. Perceived behavioral control iv. Intention
	Primary Findings	i. Intervention diminished favorable views of hookah smoking and increased awareness of physiological, psychological, and social risks associated with hookah use. ii. The intervention deterred the initial attempt to smoke, particularly among students who had never smoked hookah before. iii. Intervention reduced social pressures that promote hookah smoking and empowered participants to refuse offers from friends. iv. Prevalent of hookah smoking was 48.1% intervention group.
Bashirian, Barati, et al., 2022 [30]	Title	The effect of an educational program for hookah use prevention among high school male students: Application of the prototype willingness model
	Country	Iran
	Study Design	Randomized control trial Intervention group received educational training and reminders.
	Setting	Two high schools in Kermanshah
	Time Period	Five weeks
	Population Description	High school male students between the ages of 15 and 18 years.
	Sample Size	83 (Male)
	Outcomes	Intention and attitude toward hookah smoking
	Theory/Model/ Constructs	Prototype Willingness Model
	Primary Findings	i. The educational intervention was effective in reducing the positive attitude associated with hookah smoking and reducing the willingness to use hookah among students in the experimental group. ii. The intervention also reduced the social pressure and expectations associated with hookah smoking among study participants. iii. Again, the behavioral intentions of participants in the experiment group to smoke hookah reduced with the intervention. iv. No prevalence mentioned
Pashaeypoor et al., 2019 [31]	Title	Determinants of Intentions toward Smoking Hookah in Iranian Adolescents Based on the Theory of Planned Behavior.
	Country	Iran
	Study Design	Qualitative study (Interview and Focus group)
	Setting	Traditional coffee and entertainment locations in Tehran, Iran.
	Time Period	Not specified
	Population Description	Adolescents between the ages of 15 and 18 years old.
	Sample Size	24, Male = 14 Female = 10
	Outcome	Beliefs and behaviors about hookah
	Theory/Model/ Constructs	Theory of planned behavior
	Primary Findings	i. Participants considered hookah smoking a better alternative to cigarettes, thinking it was less harmful, which motivated them to continue using it. ii. The cultural importance of hookah and the belief in its harmlessness also promoted its use. iii. Participants believed they could stop smoking hookah at will, and it was not addictive. iv. No prevalence mentioned.
Aghdam et al., 2021 [32]	Title	Effects of a multi-level intervention on hookah smoking frequency and duration among Iranian adolescents and adults: an application of the socio-ecological model.
	Country	Iran
	Study Design	Quasi-experimental field study with intervention and control group Telegram app to exchange text messages, images, and brief videos that promote awareness of the risks
	Setting	Coffee shops in Hashtrud County for the intervention group and Qarah-Aghaj for the control group.
	Time Period	Eight weeks
	Population Description	Young adults with an average of 26 years.
	Sample Size	133 (Male)
	Outcome	Reduction of hookah use behaviors.
	Theory/Model/ Constructs	Socio-ecological Model
	Primary Findings	i. Decrease in both the frequency and duration of hookah use among participants, with no significant changes observed in the control group. ii. The intervention group's average perceived sensitivity was higher compared to the control group. iii. The average perceived severity, referring to the awareness of hookah's harmful effects, increased among the intervention group participants. iv. Furthermore, the perceived reward, or the benefits gained from hookah smoking, diminished among the intervention group participants. v. Participants reported that the perceived environmental support played a role in their decision to abstain from the behavior. vi. The prevalence of hookah smoking was ~ 72.6% in the intervention group.
Aanyu et al., 2019 [33]	Title	Prevalence, knowledge, and practices of shisha smoking among youth.
	Country	Uganda
	Study Design	Cross-sectional study
	Setting	Drinking bars in Makindye and central divisions of Kampala
	Time Period	Three months
	Population Description	Secondary and tertiary students from two divisions of Kampala with a mean age of 24.8 years.
	Sample Size	530, Male = 369 Female = 161
	Outcome	Attitude and knowledge
	Theory/Model/ Constructs	Knowledge, attitude, subjective norms
	Primary Findings	i. The research showed that students possessed sufficient knowledge regarding hookah and its detrimental health effects. ii. Students' attitudes towards hookah use turned positive when family members or friends participated in the activity. iii. Prevalent of hookah smoking is 36.4% (more males vs females)
Haroon et al., 2014 [34]	Title	Knowledge, attitude, and practice of water-pipe smoking among medical students in Rawalpindi, Pakistan.
	Country	Pakistan
	Study Design	Cross-sectional study
	Setting	Rawalpindi Medical College
	Time Period	Not specified
	Population Description	College students between the ages of 19 and 21 years.
	Sample Size	724, Male = 219 Female = 505
	Outcome	Knowledge and practices of hookah smoking
	Theory/Model/ Constructs	Knowledge and attitudes
	Primary Findings	i. Students possessed sufficient knowledge regarding hookah, including its harmful effects and addictive nature. ii. Despite acknowledging the negative health consequences, students are often persuaded by their peers to partake in smoking. iii. Prevalent of hookah smoking was ~ 22.4%
Momenabadi et al., 2018 [7]	Title	Effect of educational intervention on intention of University students' disuse of Hookah smoking: BASNEF model.
	Country	Iran
	Study Design	Randomized control trial Educational pamphlets
	Setting	Male and female students' dormitories in Kerman University
	Time Period	Eight weeks
	Population Description	The mean age of the intervention and control was 25.62 and 26.22 years, respectively.
	Sample Size	80, Male = 40 Female = 40
	Outcome	Intention and reduction of hookah smoking
	Theory/Model/ Constructs	BASNEF Model i. Behavioral intention ii. Subjective norms iii. Enabling factors
	Primary Findings	i. Educational interventions were observed on the family, roommates, and friends of the intervention group, and this was a result of the educational pamphlets and warning posters seen in the dormitory and the educational messages conveyed to the roommates and family members. ii. The attitudes and behavioral intentions of intervention students improved. iii. No prevalence
Rajabalipour, Sharifi, et al., 2019 [35]	Title	Application of social cognitive theory to prevent waterpipe use in male high-school students.
	Country	Iran
	Study Design	Quasi experimental intervention Presentation and posters
	Setting	High school in Kerman city
	Time Period	Four months
	Population Description	Male adolescents, Intervention group (n = 83), control (n = 89). Adolescents were between the ages of 15 and 17 years.
	Sample Size	172 (Male)
	Outcomes	Knowledge and quitting smoking
	Theory/Model/ Constructs	Social-Cognitive Theory
	Primary Findings	i. Knowledge of participants increased their curiosity to experiment with hookah smoking. ii. Participants with high self-efficacy were more likely to quit smoking. iii. Environmental factors did not influence the participants’ attitudes toward hookah smoking. iv. Prevalent of hookah smoking was ~ 48.5%
Abedini et al., 2014 [36]	Title	Predictors of Non-Hookah Smoking Among High-School Students Based On Prototype/Willingness Model
	Country	Iran
	Study Design	Cross-sectional
	Setting	High School in Bandar Abbas
	Time Period	Not specified
	Population Description	Participants were between 14 and 18 years old.
	Sample Size	211 Male 114 and Female 97
	Outcomes	Refraining from hookah smoking
	Theory/Model/ Constructs	Social Cognitive Theory
	Primary Findings	i. Subjective norms, willingness, and attitude accounted for 46.9% of the non-smoking hookah intention variance, with willingness being a stronger predictor than others. ii. A significant relationship between the participants’ negative prototypes about hookah smokers and their willingness to avoid hookah smoking. iii. 18% of participants had a history of smoking hookah.
Alanazi et al., 2017 [37]	Title	The Use of planned behavior theory in predicting cigarette smoking among Waterpipe smokers
	Country	Saudi Arabia
	Study Design	Cross-sectional
	Setting	Waterpipe lounges in Riyadh
	Time Period	Not specified
	Population Description	Young adults with a mean age of 26.97 years
	Sample Size	406
	Outcomes	Intention to smoke and actual cigarette smoking
	Theory/Model/ Constructs	Theory of Planned Behavior
	Primary Findings	i. Cigarette smoking and intention to smoke cigarettes were predicted by attitude and perceived behavioral control ii. Subjective norm had an indirect effect on intentions through attitude and perceived behavioral control. iii. Prevalence of hookah smoking was ~ 70.9%
Bashirian, Effatpanah, et al., 2022 [38]	Title	Investigating the factors of hookah smoking before and during the COVID-19 pandemic: Application of the Protection Motivation Theory
	Country	Iran
	Study Design	Cross-sectional
	Setting	Hamedan City
	Time Period	Not stated
	Population Description	50.7% male and 49.5% female with a mean age of 28.8 years ± 9.6 years
	Sample Size	560 people, Male = 278, Female = 285
	Outcomes	Factors affecting hookah use during the COVID-19 pandemic
	Theory/Model/ Constructs	Protection Motivation Theory
	Primary Findings	i. Perception sensitivity and self-efficacy were respectively the important predictors for the intention of hookah use behavior ii. The prevalence of hookah use decreased from 41.8% before the pandemic, to 35% during the pandemic
Dadipoor et al., 2020 [39]	Title	Predictors of Hookah Smoking among Women in Bandar Addas, Southern Iran: A Cross-sectional Study Based on the Intervention Mapping Protocol.
	Country	Iran
	Study Design	Cross-sectional
	Setting	Bandar Abbas
	Time Period	October 2018 to August 2019
	Population Description	31% of participants had a diploma degree and mean age was 31.1 ± 13.3 years
	Sample Size	332 women
	Outcomes	Predictors of hookah smoking behavior
	Theory/Model/ Constructs	Intervention Mapping Protocol
	Primary Findings	i. Attitudes, self-efficacy, habits, and intention were predictors of hookah smoking ii. Knowledge and social norms were not associated with hookah smoking iii. Prevalence of hookah smoking was 1.76 times higher in women who did not want to quit
Dehdari et al., 2022 [40]	Title	The use of theory of planned behavior variables in predicting the intention of waterpipe tobacco smoking cessation among Iranian consumers
	Country	Iran
	Study Design	Cross-sectional
	Setting	Two villages of Shiraz, Southern Iran
	Time Period	November 2016 to April 2017
	Population Description	Average age of participants was 38.36 years
	Sample Size	294 participants, Male = 127 Female = 167
	Outcomes	Predictors of intention
	Theory/Model/ Constructs	Theory of planned behavior
	Primary Findings	i. Attitude, subjective norm and perceived behavioral control had correlation to WP tobacco smoking cessation. ii. TPB variables predicted only 19.1% of the variance of behavioral intention. iii. Subjective norms had the highest weight. iv. Prevalence of hookah smoking was 7.86 times per week among participants.
Hassani et al., 2019 [41]	Title	Effect of Educational Intervention Based on Theory of Planned Behavior on the Reduction of Water Pipe Smoking in Women
	Country	Iran
	Study Design	Quasi-experimental study
	Setting	Health Centers in Bandar Abbas
	Time Period	Not stated
	Population Description	Women from the health centers with a mean age of 38.37 years
	Sample Size	128 participants. Intervention group n = 64; Control group n = 64
	Outcomes	Educational intervention on reduction of Waterpipe smoking
	Theory/Model/ Constructs	Theory of Planned Behavior
	Primary Findings	i. Mean scores of subjective norms and behavioral intentions were higher in the intervention group ii. Prevalence not mentioned.
Najafi et al., 2023 [5]	Title	Effect of educational intervention based on the theory of planned behavior on promoting preventive behaviors of oral cancer in rural women
	Country	Iran
	Study Design	Quasi-experimental study
	Setting	Rural health centers in Fasa and Shiraz
	Time Period	2021–2022
	Population Description	Female hookah users with average ages of 41.12 years and 40.63 years in the experimental and control groups, respectively
	Sample Size	120 females: 60 in the experimental and 60 in the control group
	Outcomes	Role of educational intervention in promoting preventive behaviors
	Theory/Model/ Constructs	Theory of Planned Behavior
	Primary Findings	i. Before intervention, there was no significant difference between experimental and control groups in knowledge, attitude, subjective norms, perception of behavioral control, behavioral intentions ii. After intervention, there was an increase in mean scores except for nicotine dependence. iii. iii. Prevalence information not given.
Sabzmakan et al., 2020 [42]	Title	Intention to quit water pipe smoking among Iranian women: a qualitative directed content analysis
	Country	Iran
	Study Design	Qualitative study with directed content analysis approach
	Setting	Hookah cafes, parks and homes in Tehran
	Time Period	Over 4 months
	Population Description	Women who lived in Tehran, aged 18 to 45 years old and were water pipe smokers
	Sample Size	26 women
	Outcomes	Intention to quit water pipe smoking
	Theory/Model/ Constructs	Theory of Planned Behavior
	Primary Findings	i. Women did not intend to quit water pipes in that time ii. The main contributing factors for lack of intention to quit were positive attitude, false beliefs towards hookah smoking, and having peers and family members who smoked or approved of its use. iii. Prevalence rate not reported
Sadeghi et al., 2021 [43]	Title	Health Communication Efforts to Reduce Hookah Use Among Adolescents
	Country	Iran
	Study Design	Pre- and post-intervention study
	Setting	Schools in Sirjan city, Kerman province
	Time Period	Not stated
	Population Description	Adolescents with mean age of 14.3 years; 131 participants were male.
	Sample Size	280 adolescent school children, Author did not categorize into gender
	Outcomes	Reduction of hookah use
	Theory/Model/ Constructs	Knowledge, Attitude and Practice Model
	Primary Findings	i. Prevalence among those who had ever consumed decreased from 8.9–4.3% and for those who used occasionally, it decreased from 35–19.6% ii. Significant change in scores of knowledges, attitude and practice after the HCE. iii. Windshield survey after intervention showed that the number of people smoking hookah decreased iv. Prevalence of hookah smoking was 44.3% among the adolescents

### Prevalence of Hookah Use Behaviors

The overall pooled prevalent rate of hookah smoking among participants was 26.4% (pooled proportion = 0.2639, SE = 0.371), indicating a significant number of young adults continue to engage in risky behavior. Twenty-two studies reported varying prevalence rates of hookah smoking, ranging from a low of 2.6% [19] to a high of 89.4% [28]. Specifically, six studies reported the highest prevalence rates, ranging from 64 to 89.4%; seven studies reported moderate prevalence rates, ranging from 35 to 48.5%; and nine studies reported lower prevalence rates, ranging from 2.6 to 32.3%.

### Study Design and Outcomes

The reviewed articles utilized various study designs to investigate hookah-related behaviors and outcomes. Fourteen studies employed a cross-sectional design to predict diverse outcomes, such as the intention to use hookah [18, 27, 40, 37], actual hookah use behavior [26, 39, 24, 20, 21, 19], attitudes and knowledge about hookah use [34, 33], quitting hookah smoking behavior [36], and factors influencing hookah use during the COVID-19 pandemic [38]. Additionally, 12 intervention studies addressed behaviors or outcomes such as increasing the intention to quit or reduce hookah smoking [7, 22, 29, 30], preventing or quitting actual hookah smoking [5, 43, 25, 28, 32, 35, 41], and preventing oral cancer [23]. Two qualitative studies explored participants’ beliefs and behaviors regarding hookah use [31] and their intentions to quit hookah smoking [42].

### Theories and Models

Seven studies utilized the full constructs of the Theory of Planned Behavior (TPB) [5, 27, 31, 34–42, 37] while four studies applied the complete constructs of Social Cognitive Theory (SCT) [25, 29–36]. Two studies used protection motivation theory [43, 38], and another two used the Prototype Willingness Model [18, 30]. Other theoretical frameworks included PRECEDE-PROCEED [23], the Multi-Theory Model [28], the Intervention Mapping Protocol [39], the Health Action Process Approach [22], and the BASNEF Model [7]. Additionally, seven studies did not explicitly identify a specific theory but integrated constructs from TPB (e.g., subjective norms and attitudes) and SCT (e.g., knowledge and environment) in their analyses [19, 20, 21, 24, 26, 34, 33]. The constructs from the theories provided the facilitators and barriers of the hookah use behavior among the participants.

### Facilitators of Hookah Smoking Behaviors

Facilitators of intention to use hookah or engage in hookah smoking behaviors include constructs from the Theory of Planned Behavior (TPB) and Social Cognitive Theory (SCT) and some constructs from health beliefs models. TPB constructs included subjective norms (i.e., family and friends influences) [18, 26, 27, 36, 42, 38–33] and participants’ favorable attitude toward hookah (i.e., positive perceptions of hookah smoking, such as finding it enjoyable or socially appealing) [27, 39, 40, 42, 37]. SCT constructs included environment (i.e., (availability, affordability, and proximity) [31, 40–33], and knowledge or awareness (i.e., limited understanding of the health risks associated with hookah use) [19, 33, 34]. The Health Beliefs Model (HBM) construct was perceived harmlessness (i.e., belief that hookah smoking is less harmful, less risky, safer, or a better alternative to cigarettes or non-addictive) [18, 31, 39, 42, 20]. Other constructs include entertainment and curiosity (i.e., The appeal of trying hookah as a leisure activity or out of curiosity) [26].

### Protective Factors of Hookah Smoking

On the other hand, TPB constructs such as attitude, subjective norms, and perceived behavioral control were found to be protective factors for non-hookah smoking behavior or prevention of hookah smoking attitude [40]. Other protective factors for non-hookah smoking behavior included SCT constructs such as self-efficacy [27, 38, 39], and HBM construct like perceived susceptibility, fear and severity [43], and awareness of harmful effects [43].

### Intervention Effectiveness

Regardless of the constructs of theory or models used, most of the intervention studies reported a significant increase in intention to quit hookah smoking [7, 22, 29, 30]. In contrast, others reported an increase in actual hookah smoking quitting behavior [5, 43, 25, 28, 32, 35, 41] and a significant reduction in the frequency of hookah smoking [29]. However, one intervention study reported no significant changes in hookah smoking [35].

## Discussion

This scoping review synthesized theory-based studies that explored hookah use behaviors among young adults in LMICs. The key findings of the review can be summarized in four main areas, including the prevalence of hookah use among young adults, various behavioral outcomes, facilitators and barriers to hookah use, and descriptive of the intervention effectiveness.

### Prevalence of Hookah Use

The prevalence of hookah smoking behavior is the first observation worth discussing: This scoping review revealed the pooled prevalence rate of 26.4%, providing a more generalized perspective on hookah use among young adults in the reviewed studies. This finding suggests that a significant portion of the studied population engages in this hookah-smoking behavior. A closer look at reviewed studies revealed a wide range of prevalence rates for hookah smoking behaviors, reflecting the diverse settings, populations, and methodologies employed. Twenty-two studies reported prevalence rates that spanned from as low as 2.6% [34] to as high as 89.4% [27]. Notably, six studies documented the highest prevalence rates, which fell between 64% and 89.4%, indicating a significant presence of hookah use in certain populations or regions. The high prevalence rates observed in some studies (64–89.4%) could be attributed to cultural norms, social acceptability, or the accessibility of hookah in those regions [44–46]. In contrast, the lower prevalence rates (2.6–32.3%) might reflect less social acceptability, stricter regulations, or lower availability in certain communities or countries. This finding aligns with global trends indicating that hookah smoking is gaining popularity, especially among younger demographics, due to perceptions of it being a safer alternative to cigarette smoking [47, 48]. However, the substantial heterogeneity in prevalence rates emphasizes the importance of contextual factors, such as socioeconomic status, cultural attitudes, regulatory environments, and targeted educational efforts. The wide range in prevalence also points to methodological variations across studies, including differences in sampling methods, study settings, and definitions of hookah use. Future research should aim to standardize these methodologies to enhance comparability and better inform public health strategies. With the significant prevalence of hookah smoking behavior, continuous efforts need to be made to address the early onset of smoking behavior, as hookah can also be addictive.

### Study Design and its Related Behavior Outcomes

Another point of interest is the nature of the study designs and behavior outcomes addressed in those reviewed studies. The reviewed studies employed diverse methodologies to investigate hookah-related behaviors and associated health outcomes, reflecting a multifaceted approach to understanding and addressing this public health issue. Cross-sectional studies were the most commonly used design, with 14 cross-sectional studies examining a range of outcomes. These outcomes included predicting the intention to use hookah, actual use behaviors, attitudes, and knowledge about hookah smoking, as well as factors influencing usage during unique contexts like the COVID-19 pandemic. Evaluating these behavior outcomes is important as they provide target intervention areas to address. While cross-sectional studies offer valuable snapshots of behaviors and predictors, their inability to establish causality limits the strength of their conclusions [49]. However, these studies collectively highlight the key range of factors (i.e., constructs from different theories) that either facilitate or deter hookah smoking behaviors. Two qualitative studies provided in-depth perspectives on beliefs, motivations, and intentions regarding hookah use and cessation. These studies enriched the quantitative findings by highlighting nuanced barriers to quitting, such as addiction, social reinforcement, and misconceptions about health risks. An important health outcome that was overlooked in the reviewed studies is the potential association between hookah use and cancer. This represents a missed opportunity to explore a critical public health concern. Future research should investigate the relationship between hookah smoking and cancer risks to address this significant gap in the literature. Such studies could provide valuable insights into the long-term health impacts of hookah use, including its carcinogenic potential, and inform targeted prevention and intervention strategies.

### Facilitators and Barriers

This study identified several factors that either facilitate or deter hookah use, particularly focusing on constructs from the TPB, SCT, and HBM frameworks. These findings underscore the complex interplay of subjective norms, attitudes, and environmental factors in shaping hookah use behaviors.

### Subjective Norms as a Facilitator and Protective Factor

The construct of subjective norms, defined as the influence of family, friends, and significant others exert on individuals’ attitudes toward hookah smoking, emerged as both a protective and facilitating factor. Dehdari et al. study [40] reported that social norms played a protective role against participants’ smoking behavior. Participants explained that disapproval of hookah use by family members and friends significantly deterred them, reducing their likelihood of engaging in the behavior. Lugemwa’s study agreed with this finding, showing that participants’ desire to quit smoking behavior was associated with family disapproval of waterpipe use [50]. In health-conscious environments or among religious or cultural groups that discourage tobacco use, individuals may refrain from smoking hookah due to the fear of social judgment or exclusion [51].

Conversely, several of the reviewed studies found that subjective norms such as peer pressure, family influence, and social acceptance were consistently key drivers behind the initiation and continuation of hookah smoking [17, 25, 26, 35, 41, 37–19]. Other studies affirmed that subjective norms were associated with the adolescents’ increased use of hookah [52–55]. This is particularly relevant in contexts where hookah smoking is normalized or even celebrated in social gatherings [53, 56]. The influence of close social circles—friends and family—plays a pivotal role in introducing individuals to hookah and reinforcing its use over time. Intervention strategies, addressing the power of social norms and peer influence could prove essential in altering perceptions and behaviors related to hookah use.

### Attitudes as a Facilitator and Protective Factor

#### Negative Attitudes

Attitudes toward hookah also presented a dual role as facilitators and barriers. One of the articles found that participants with negative attitudes toward smoking were the key factors in discouraging hookah use [39]. These negative attitudes create a deterrent by reinforcing the idea that smoking is harmful or unacceptable, a conclusion also supported by Sidani et al. [57].

#### Positive Attitude

However, participants’ positive attitudes, such as viewing hookah as a socially appealing, enjoyable, or recreational activity, emerged as significant facilitators in many of the reviewed studies [26, 38, 39, 41, 20]. Other studies supported this finding that when participants had positive attitudes and perceived hookah smoking as a fun, recreational, or less harmful alternative to other forms of tobacco use, it encouraged its usage [17, 55, 58, 59]. This finding underscores the importance of shifting public perceptions in health campaigns, aiming to alter how hookah is perceived socially and personally. Changing attitudes through targeted education about the risks and consequences of hookah smoking could reduce its appeal.

### Other Facilitators of Hookah Smoking

Several additional factors facilitated hookah use, including misconceptions about its health risks, environmental factors, lack of knowledge of the harmful effects or underestimating the harmful effects of the hook, smoking hookah out of curiosity or as a source of entertainment, and the beliefs that hookah is non-addictive.

### Beliefs that Hookah is Harmless

A common misconception among participants was that hookah smoking is relatively harmless, less risky, or even safer than cigarettes [17, 30, 38, 41, 24]. Other studies align with our reviewed findings, demonstrating that the misconception that hookah use is harmless significantly influences individuals’ decisions to start or continue smoking [60–62]. This finding suggests that participants may not fully understand or acknowledge the associated health risks. Educating the public about the dangers of hookah use, including the risks of cancer, respiratory disease, and other long-term health consequences, should be a priority in intervention strategies. By dispelling the myth of hookah as a safe alternative, health campaigns can better convey the importance of avoiding all forms of tobacco use.

### Environmental Factors

The environmental context, including the affordability, accessibility, and proximity of hookah-smoking venues, was found to facilitate hookah use [30, 39–19]. Hookah smoking is often linked to social spaces such as coffee shops, bars, or hookah lounges, which may make it easier for individuals to engage in the behavior. Reducing the availability of these environments or regulating their hours of operation could effectively limit access to hookah. Furthermore, targeting policy changes that restrict the sale or use of hookah in public spaces might help decrease overall prevalence.

### Lack of Knowledge

While some participants in the reviewed studies had a basic understanding of hookah smoking, many were unaware of its health risks [40–19]. This knowledge gap indirectly facilitated continued use, particularly among youth and young adults. Educational interventions aimed at increasing awareness of the dangers of hookah smoking are critical. These could include targeted campaigns in schools, universities, and public spaces to emphasize the risks of cancer, respiratory diseases, and other health consequences associated with hookah use.

### Entertainment and Curiosity

Hookah smoking was often viewed as a recreational activity driven by curiosity and the social experience [25]. Many young people were initially attracted to hookah smoking out of a desire to fit in, try something new, or for the novelty of the experience [17, 46, 63]. This finding suggests that public health campaigns should not only focus on health risks but also address the social and cultural aspects of hookah use [64]. Creating alternative social activities or environments that offer similar social rewards without the harmful effects of hookah smoking could serve as a deterrent.

### Perceived Non-Addictiveness

Some participants believed that hookah smoking was not addictive or that they could quit at will, which facilitated continued use. This belief in the non-addictiveness of hookah may be tied to the perception that it is safer or more controllable than other forms of smoking [65]. Interventions that educate individuals about the addictive nature of nicotine and the potential for hookah smoking to lead to dependency should be a priority. Highlighting the parallels between hookah smoking and cigarette smoking in terms of addiction and long-term health effects could be effective in countering this misconception [66, 67].

### Other Protective Factors Against Hookah Use

#### Self-Efficacy

Confidence in one’s ability to resist peer pressure and avoid smoking hookah was identified as a key protective factor [26, 36, 38]. Self-efficacy, or the belief that one can control their behavior and make healthy choices, is an important determinant of smoking cessation [68]. Programs that enhance individuals’ self-efficacy through skill-building exercises, goal-setting, and supportive environments could help them resist the temptation to start or continue hookah smoking.

#### Perceived Risk and Fear

Awareness of personal susceptibility to harm and fear of health consequences from hookah smoking were deterrents for some participants [23]. Similarly, other studies found that awareness of harmful effects, such as irreversible lung damage and cancer, evoked fear among participants, discouraging tobacco use [69, 70]. These findings align with the conclusions of our review. Interventions that emphasize the severity of the health risks, such as lung disease, cancer, and other long-term effects, may be effective in motivating individuals to avoid or quit hookah smoking. However, these interventions must be balanced with empowerment strategies that offer support and solutions rather than simply instilling fear.

#### Intervention Effectiveness

Twelve intervention studies focused on reducing hookah smoking prevalence, increasing quitting intentions, and preventing associated health risks like oral cancer. Most interventions applied theoretical frameworks such as the TPB, SCT, HBM, and other related constructs. Despite the differences in the specific theories and models utilized, the majority of the studies demonstrated a significant increase in the intention to quit hookah smoking. For instance, studies using TPB [7, 18, 28, 29] showed that participants who were exposed to interventions had greater intentions to quit smoking hookah, likely due to the influence of enhanced attitudes, subjective norms, and perceived behavioral control. In addition to this intention, other studies reported tangible outcomes in terms of actual quitting behavior. Specifically, interventions aimed at cessation of hookah smoking led to increases in the number of participants successfully quitting, as seen in studies conducted by [5, 23, 43, 27, 31, 32, 40]. Additionally, some studies noted a significant reduction in the frequency of hookah smoking, even when cessation was not fully achieved, suggesting that interventions might help reduce the overall amount of hookah use in certain populations [28]. Successful interventions were characterized by targeted strategies addressing self-efficacy, perceived risks, and social norms. The findings from the reviewed intervention studies underscore the potential efficacy of behavioral interventions in reducing hookah smoking behaviors, with most studies reporting positive outcomes. These outcomes are in line with existing literature that suggests interventions based on behavioral theories, such as TPB and SCT, can effectively address attitudes, intentions, and behaviors related to smoking cessation [71, 72].

However, it is important to note that not all intervention studies produced favorable results. One study [32] reported no significant changes in hookah smoking behavior despite its intervention efforts. The mixed results of the interventions underscore the importance of tailoring approaches to cultural and contextual factors, particularly in LMICs. This finding raises questions about the factors that may influence the success or failure of such programs, such as the intensity or duration of the intervention, participant engagement, or the contextual factors influencing hookah use in the specific population.

### Limitations and Strengths

This scoping review has several limitations that should be considered when interpreting the findings. A significant limitation of this review is its reliance on the description of the included studies. While the review synthesizes the authors’ findings on intervention outcomes and behaviors related to hookah use, it does not evaluate the methodologies, study settings, sample size calculations, or inclusion and exclusion criteria used in the reviewed studies. This focus on describing rather than critiquing the intervention methodologies limits the ability to assess the rigor and generalizability of the findings. However, this review emphasizes synthesizing practical insights into hookah-related behaviors and intervention outcomes. Another limitation is the lack of evaluation of the validity and reliability of the instruments used in the cross-sectional studies. Many of these studies relied on self-reported data, which can be influenced by social desirability bias and recall errors. Without assessing the psychometric properties of the tools used to measure variables such as attitudes, knowledge, and intentions, the accuracy and consistency of the reported findings remain uncertain. However, this scoping review provides the breadth and range of existing literature on a topic rather than critically appraises individual study quality or measurement tools. This review focuses on studies grounded in behavioral theories, which introduces potential challenges. For example, the application and operationalization of theoretical constructs may vary significantly across studies, leading to inconsistencies in how key factors such as self-efficacy, attitudes, and subjective norms are defined and measured. Additionally, scoping reviews are inherently broad in scope and do not critically appraise the quality of included studies. This approach, while useful for mapping the existing literature, may overlook nuanced methodological weaknesses and variations in how theories are applied.

#### Strengths

This scoping review has several notable strengths that enhance its contribution to the understanding of hookah-related behaviors and interventions, particularly among young adults in LMICs. One of the key strengths of this review is its focus on synthesizing theory-based studies. By highlighting the use of behavioral theories, this review provides valuable insights into the theoretical underpinnings of hookah-related behaviors and interventions. This approach helps bridge the gap between theoretical constructs and practical applications, offering guidance for designing future interventions. The emphasis on studies conducted in LMICs addresses a critical gap in the literature, as these regions often experience unique cultural, social, and economic factors that influence hookah use. Focusing on LMIC settings provides contextually relevant findings that can inform targeted strategies for addressing hookah smoking in vulnerable populations. This review captures a wide range of hookah-related behaviors and outcomes, from smoking prevalence and predictors to intervention outcomes and cessation efforts. This broad scope allows for a more comprehensive understanding of the factors driving hookah use and the effectiveness of various interventions, offering a holistic perspective on the issue. The use of a systematic and transparent search strategy across multiple databases ensures the inclusion of diverse studies, increasing the generalizability of findings. Furthermore, employing predefined inclusion and exclusion criteria and utilizing tools such as Covidence for screening and data extraction enhances the reliability of the review process. By mapping the existing literature, this review identifies critical gaps, such as the need for more rigorous study designs and validated measurement tools. In summary, this scoping review provides a robust and well-rounded synthesis of theory-based studies on hookah use among young adults in LMICs, offering important insights for researchers, practitioners, and policymakers.

### Implications

Future research should consider conducting systematic reviews or meta-analyses to critically appraise and synthesize the quality of evidence in this area. Evaluating the validity of measurement tools and the fidelity of theoretical applications would strengthen the understanding of hookah-related behaviors and interventions. Moreover, refining inclusion criteria to focus on high-quality, theory-based research may provide more robust insights into effective strategies for reducing hookah use.

The findings emphasize the need for integrated strategies combining robust behavioral frameworks with culturally sensitive interventions. Future research should explore longitudinal designs to establish causal relationships and assess the sustained impact of intervention strategies. Addressing individual and environmental factors is crucial for reducing hookah use prevalence and mitigating its health consequences.

## Conclusion

This scoping review highlights the growing body of literature on hookah smoking behaviors and interventions among young adults in LMICs, with a specific focus on studies utilizing behavioral theories. The findings reveal significant variations in prevalence rates, key predictors of hookah use, and the effectiveness of interventions aimed at reducing smoking behaviors. The application of theories such as the Theory of Planned Behavior (TPB) and Social Cognitive Theory (SCT) underscores the importance of understanding behavioral drivers and leveraging theoretical frameworks to design targeted interventions.

While the review demonstrates the potential of theory-based approaches, it also identifies critical gaps, including the need for more rigorous study designs, culturally sensitive interventions, and validated measurement tools. These limitations highlight the importance of future research to refine intervention strategies and evaluate their long-term effectiveness in reducing hookah use and its associated health risks.

By synthesizing evidence on hookah smoking behaviors and interventions, this review offers valuable insights for researchers, practitioners, and policymakers. It emphasizes the need for comprehensive, theory-driven approaches to address individual, social, and environmental factors influencing hookah use. Additionally, it calls for stronger policies and educational efforts tailored to the unique challenges faced in LMICs. Addressing these gaps will be crucial for reducing the prevalence and health impacts of hookah smoking in these vulnerable populations.
